# Animal Welfare Assessment in 16 Zoos in South Korea Using the Modified Animal Welfare Assessment Grid

**DOI:** 10.3389/fvets.2022.860741

**Published:** 2022-04-26

**Authors:** Seung-Aee Ma, Hye-Jin Kang, Kyuyoung Lee, Sun-A. Kim, Jin Soo Han

**Affiliations:** ^1^Department of Laboratory Animal Medicine, Institute for the 3Rs & Animal Welfare, College of Veterinary Medicine, Konkuk University, Seoul, South Korea; ^2^Center for Animal Welfare Research (CAWR), College of Veterinary Medicine and Research Institute for Veterinary Science, Seoul National University, Seoul, South Korea; ^3^University of California-Davis School of Veterinary Medicine, Davis, CA, United States; ^4^Veterinary Teaching Hospital, College of Veterinary Medicine, Chungbuk National University, Cheongju, South Korea

**Keywords:** zoo animals, animal welfare, zoo animal welfare, zoo welfare assessment, animal welfare assessment grid, South Korea

## Abstract

Various assessment tools that have been proposed thus far have disadvantages in that they are complex, time-consuming, non-objective, and not convenient for assessing multiple zoos. This study aimed to develop a simple, objective, and reliable welfare assessment tool, the modified Animal Welfare Assessment Grid (AWAG), that can be applied in South Korea, where there is no licensing system for zoos. The AWAG has four main sections: physical, psychological, environmental, and procedural. These four sections include 23 welfare factors like general conditions, behaviors, housing, and restraints, for which each individual or group of animals is given a score. The modified AWAG system was applied by converting the 10-point rating scale of the original AWAG to a 6-point Likert scale. Sixteen zoos in Korea were selected based on the zoos with the most animals. Three inspectors assessed the scores of each animal and then averaged the results. The total data surveyed included 16,065 items. Zoos were largely classified into four grades based on the size of the zoo, animal species, and operating organization. In a relatively short period of 14 days, all the zoos were successfully assessed. Despite the shortened and modified assessment tool, the inter-rater reliability among inspectors was 0.942 with high objectivity. The modified AWAG could identify welfare differences between grades of Korean zoos. There were large differences between zoos in most environmental sections and some zoos were evaluated as having inadequate welfare levels. The modified AWAG showed high usability and objectivity. In addition, it was possible to determine which environmental or procedural sections could potentially help improve physical and psychological scores. The modified AWAG is an objective method that could set the direction for the improvement of zoo welfare in the future.

## Introduction

Modern zoos are expected to enhance the welfare and physical and mental well-being of animals (1). Public interest in zoo animals demands higher welfare from individual zoos as society evolves, and failure to provide sufficient welfare could threaten the validity of a zoo's existence.

Many countries regulate the welfare standards of zoos by law. Countries that are active in animal protection, such as the United Kingdom, France, and Australia, inspect and regulate the welfare status of zoos through licensing systems. Although they vary between countries, the standards in zoo licensing systems usually include minimum requirements for animal care. In Brazil, France, and Switzerland, the legislation includes cage size, while basic requirements are included in the Philippines. Australia, the UK, India, New Zealand, and the United States have their own legislative standards. The most detailed are the voluntary standards in the US, Canada, and Europe. These standards are monitored by inspection (2).

In fact, most inspections are carried out by inspectors through document reviews, field investigations, and inspection reports in many countries (3) Some countries, such as Switzerland, request quantitative standards such as cage size; but the other countries, such as the UK, the welfare inspections are generally conducted comprehensively as if answering standard questions: for example, 'are the animals provided with a high standard of nutrition?' with responses including yes, no, or N/S (4). Therefore, the inspection of the zoo may vary depending on the quality of the inspector.

Modern zoos in South Korea began in 1909 in Chang-gyeong-won. Daegu Dalseong Zoo, Seoul Children's Grand Park, and Natural Farm (currently Samsung Everland) opened in the 1970s (5). According to the Ministry of Environment, a total of 107 zoos in Korea were registered in April of 2021 (6). However, as animal welfare issues in zoos have emerged socially, such as the influx of animal cafe-type indoor zoos, criticism of zoos by animal rights groups has been increasing. The Korean government enacted the Act on the Management of Zoos and Aquariums in 2016 (7). Contrary to expectations, however, this law allows zoos to operate without specific restrictions, which raised concerns regarding animal welfare; thus, a permit system based on the UK zoo license system was proposed. However, this system depends on subjective inspections, the reliability of which is suspicious due to the lack of inspectors with sufficient knowledge and experience in Korea. Therefore, there is a need for a reliable, objective, and efficient method for evaluating animal welfare that can be used to evaluate zoos.

Animal welfare is measured on a continuous scale from good to bad. Many studies have evaluated animal welfare (8). Although farm animal-based assessments were initially used (9), researchers have developed zoo-animal-specific welfare assessment tools (8, 10–13). Animal welfare assessments were initially conducted as surveys to which the staff responded (Table 1) (14, 15). An inspector-evaluation-based animal welfare assessment was then conducted, targeting a small number of species (Table 1) (11, 12, 16). In addition, more schematic and objective assessment tools have been proposed (Table 1) (10, 13, 15, 17). However, these tools have some limitations. These previous studies questioned the measurement of the welfare level of each zoo using a step-by-step scoring system. The exact criteria between scores may be ambiguous, resulting in differences in scores. In other words, these rating systems rely on subjective evaluations; hence, if the evaluator changes, the scores may also change.

**Table 1:** Literatures on animal welfare assessment.

**Year**	**Authors**	**No. of institutions**	**Species/subject**	**Method**	**Scale**	**Time**	**Inspectors**	**Reference**
2001	Bashaw et al.	49	214 Giraffe, 29 okapi	Stereotypic behavior-based	16-item Survey		Familiar staff response by mail	(9)
2009	Cho et al.	12	Mammals (total)	5 domains based 68 questions	survey		Internal zoo staff (over 3 years)	(18)
2015	Clegg et al.	3	20 Bottlenose dolphins	36 species-specific measure C-Well®	0, 1, 2 of 0~5	2 days for 10 dolphins	Team of specialist 4 DVM, 3 welfare PhDs, 2 curators	(11)
2015	Kagan et al.	a number of zoos.	Institution and animal/environment	four major components:	4 steps Yes, Somewhat, No, Not clear		3 perspectives-internally, familiar external, external	(6)
2017	Wolfensohn et al.	2	Primates group, 17 Avian species	4 sections	1~10 score	95 days	Zoo staff, animal care staff, DVM, welfare advisor	(14)
				23 factors				
2018	Fersen et al.	1	2 Bottlenose Dolphins, Antillean Manatees	4-step decision Tree survey	4 steps: 1~2, 3~4, 5~6, 7~8		Step 1: institution	(7)
							Step 2: official veterinarian	
				→ theoretical Analysis			Step 3: Zoo and Official Veterinarian	
							Step 4: inspector	
				→ In situ Inspection				
				→ conductive report				
2018	Sherwen et al.	3	339 species 628 assessment	5 domains based 20 indicators(15 resource-based welfare risk factors and 5 animal-based measures)	0, 1, 2	3 years	Experienced zoo staff	(8)
2020	David J. Mellor et al.		Farm animals and companion animals, ferrets, stoats, weasels, kangaroos, wallabies, possums, cetaceans, reptiles, amphibians and fish.	5 domains:1 Nutrition, 2 Physical Environment, 3 Health, 4 Behavioral Interactions and 5 Mental State	5 scale of Quality of life, 4~5 scales in Human interaction		scientifically informed experts	(10)

The Association of Zoo and Aquarium (AZA) Animal Welfare Committee also distributed guidelines for animal welfare evaluation to member organizations, broadly presenting input factors, positive output factors, and negative output factors for items such as nutrition, environment, health, behavior, choice, control, and mental state (18). However, these guidelines only suggested which aspects of animal welfare should be evaluated and encouraged each institution to develop its own evaluation methods (19). To date, objective evaluation of animal and institutional welfare is lacking in AZA-accredited facilities. Moreover, no validated assessment method exists to compare species and institutions, and zoo animal welfare assessment remains complex (20). In addition, to determine which zoo's welfare situations and management are appropriate, individual measurements must be performed differently for each animal in the zoo, making the evaluation more challenging. This is particularly true because zoos, unlike farms, contain at least 10,000 animal species (21). In addition, it is prohibitively time-consuming to find and apply all the appropriate measurement methods.

The measurement method proposed by Justice et al. (2017), the Animal Welfare Assessment Grid (AWAG), minimizes subjective evaluation (22). This evaluation method has the most distinct criteria among the latest studies and takes the form of a quantitative measurement system with little difference between inspectors (Table 1) (22). This measurement tool divides animal welfare into four sections—physical, psychological, environmental, and procedural—and is reported as a percentage or distinct standard for each item on a 10-point scale. These scales allocate detailed scores based on the state of welfare for each point. For example, a score of 3 for the general condition score in the physical section represents “weight outside normal range by <10%,” which is an objective value that cannot be interpreted differently between inspectors. Therefore, the AWAG system is more objective than methods that used more vague classifications like “good/bad” or numeric 5-point scales. A more objective welfare evaluation system will help to improve the quality of life of animals, as an animal appearing to be in pain by subjective human measures is not necessarily in pain (23). In addition, the reliability of the evaluation method can be ensured only when similar results are obtained, particularly when measured by several people in multiple zoos. In addition, animal welfare assessments are time-consuming; thus, methodological improvements and adjustments are required.

The various assessment tools that have been proposed thus far have disadvantages in that they are complex, time-consuming, subjective, and inconvenient for assessing multiple zoos (Table 1). In addition, a limited number of animal welfare assessments have been conducted in Korean zoos. Therefore, this study aimed to develop a simple, objective, and reliable welfare assessment tool, the modified AWAG, which can be applied to multiple Korean zoos without a licensing system.

## Materials and Methods

### Research Period

From June 15, 2020 to July 15, 2020, 16 zoos in Korea were surveyed over 14 days. This study investigated the welfare level of Korean zoos, as requested by the Ministry of Environment of Korea. Local government officials in charge of zoo management and the National Institute of Ecology notified each zoo in advance and requested their cooperation.

### Inspectors

The survey was conducted by three inspectors: one veterinarian (22 years of zoo experience), one zookeeper (one with 35 years of experience with mammals and birds or one with 20 years of experience with reptiles), and one animal welfare researcher (9 years of experience with welfare assessment). The veterinarian, zookeeper with experience with mammals and birds, and animal welfare researcher visited all 16 zoos to conduct every assessment. The zookeeper with experience in reptiles conducted assessments at zoos with reptiles.

### Welfare-Assessed Zoos

As of March 2021, 107 zoos have been registered in Korea. The original AWAG system is a 10-point rating scale (17). This study modified the scale to a 6-point Likert scale and assessed its practicality and efficiency. According to 2019 survey data of the Ministry of Environment, the zoos were classified into four grades based on their size, number of animal species, and operating organization. Grade A zoos (A1 and A2) are globally accredited zoos, such as by the AZA. Grade B zoos (B1–B4) are nationally certified by the Korean Association of Zoos and Aquarium (KAZA) (24). Grade C zoos (C1–C4) are characterized by large zoos with ≥ 50 species, ≥1,000 individuals, and ≥3,000 m^2^. Grade D zoos (D1–D6) are the remaining small uncertified zoos, such as petting zoos. This study assessed at least 10% of zoos from each grade. A total of 16 zoos were selected based on the zoos with the most animals (Table 2).

**Table 2:** Grade of 16 assessed zoos in South Korea.

**Zoo grade**		**Zoos**	**No. of zoos**	**% of zoos**	**Assessed zoos**	**Ratio**
**A**	Globally accredited zoos (AZA, EAZA etc.)	A1, A2	2	2	2	100%
**B**	Nationally certified zoos (nationally recognized zoo associations: KAZA)	B1, B2, B3, B4	11	10	4	36%
**C**	Large uncertified zoos, ≥50 species, ≥1,000 individuals, ≥3,000m^2^	C1, C2, C3, C4	38	35	4	10%
**D**	Small uncertified zoos, <50 species, <1,000 individuals, <3,000m^2^	D1, D2, D3, D4, D5, D6	56	51	6	10%
	Closed zoos		2	2	0	
**Total**	109	100	16	

*AZA, Association of Zoos & Aquariums: developed 48 years experienced accreditation to keep gold standard; KAZA, Korean Association of Zoos & Aquariums: Registered with the Ministry of Environment and the most validated association in Korea*.

### Animals

The zoo animal registration data from the Korean Ministry of Environment were categorized into 11 groups. Among these groups, 11 species— prairie dogs (*Cynomys ludovicianus*), rabbits (*Oryctolagus cuniculus)*, Japanese macaques (*Macaca fuscata)*, raccoons (*Procyon lotor)*, fennec foxes (*Vulpes zerd)*, meerkats (*Suricata suricatta)*, tigers (*Panthera tigris)*, macaws (*Ara ararauna* or *A. chloropterus*), cockatiels (*Nymphicus hollandicus)*, pythons (*Python regius* and *Python bivittatus)*, and African spurred tortoise (*Centrochelys sulcata*)—were selected from each group to include as many species as possible in the in the 16 institutions targeted for assessment. This study included a total of 153 animals.

### Research Methods

The AWAG scoring system proposed by Justice et al. (2017) has four main sections: physical, psychological, environmental, and procedural (22). The same indicators were scored in the form of percentages or distinct standards. The physical section has five factors: general condition (weight, body condition score, and feather condition), clinical assessment (injury, feather damage/alopecia, and vomiting), fecal consistency, activity level (mobility), and food and water intake (hunger and thirst). The psychological section has six factors: abnormal behaviors (regurgitation, stereotypy, and automutilation/feather plucking), responses to catching events, social disruption within groups (levels of aggression or bullying, as well as the duration), enrichment provision and use, aversion to routine events, and animal training. The environmental section has six factors: housing, group size, enclosure furnishings, nutrition (requirements of both individuals and species), access, and contingent events. The procedural section has six factors: restraint, sedation/anesthesia, time a bird is restrained before/during the procedure, veterinary procedure, change in daily routine, and visitor score (Supplementary Tables 1–4). The present study modified the AWAG scoring system to use a 6-point scale arranged in the order of “very good” to “worst”, as follows: 1, 3 (combined 2 and 3), 5 (combined 4 and 5), 7 (combined 6 and 7), 9 (combined 8 and 9), and 10. Three inspectors assessed the scores for each animal and averaged the results. The inspectors visited each animal's enclosure with the zookeeper. Before starting the assessment, the animal care records of each zoo were reviewed. Each factor was assessed individually by observing each animal for at least 30 min. The inspectors recorded each factor score using their own mobile device or tablet and performed statistical processing using Google Forms (Google, Mountain View, CA). Total assessment times of approximately 6–8 h and 4–5 h were required for large zoos and small petting zoos, respectively. The inspectors discussed only the observation results, such as checking the number of injured animals that could not be observed by the other inspector. The rating was performed blindly using their own device. The survey data included 16,065 items (153 types ×3 inspectors ×35 items).

### Descriptive Statistics

Descriptive statistics were used to summarize the 23 AWAG scores of 11 species from 16 zoos measured by three inspectors (veterinarian, zookeeper, and researcher) using frequency tables and graphs.

### Inter-rater Reliability of Modified AWAG

This study evaluated the inter-rater reliability (IRR) of AWAG scores among the veterinarians, zookeepers, and researchers. Fleiss' kappa was measured to evaluate the IRR of AWAG scores among all three inspectors. A linear-weighted Cohen's kappa was used to measure the IRRs of each pair of inspectors. These assessments were performed using the “Irr” package in R (R Foundation, Vienna, Austria) (25).

### Statistical Differences in Modified AWAG Scores by Zoo Level

We evaluated the statistical differences in AWAG scores by zoo level using Kruskal–Wallis analysis of variance (ANOVA). We first calculated the mean of the three inspectors' scores (23 scores ×153 individual animals) and each AWAG section score (physical, psychological, environmental, and procedural) to determine the statistical significance of the differences between AWAG scores by zoo level (four categories ×153 individual animals). Kruskal–Wallis ANOVA was used to evaluate the statistical differences in the four averaged AWAG scores between the four zoo levels in the Republic of Korea (α=0.05). We additionally performed pairwise comparison tests using Wilcoxon rank-sum tests with Benjamin and Hochberg's *p*-value corrections and Tukey's tests to determine the statistical significance in the difference in scores for each pair of the four zoo levels (α = 0.05) (26).

### Associations of Physical and Psychological Scores With Environmental and Procedural Scores

Multivariable linear regression was used to evaluate the association of each physical and psychological score with the six environmental and six procedural scores (α = 0.05). We averaged each of the 23 scores measured by the three inspectors for the regression analysis. Backward stepwise selection was used to determine the association of each of the five physical and six behavioral scores with the six environmental and six procedural factors based on the Akaike information criterion. After determining the final models of the 16 physical and psychological scores, we summarized the odds ratios of the significant factors and their 95% confidence intervals. All descriptive statistics, IRRs, Kruskal–Wallis ANOVA, multivariable linear regression, and graphic visualizations were performed using R software (R Foundation) (27).

## Results

**Figure 1:**
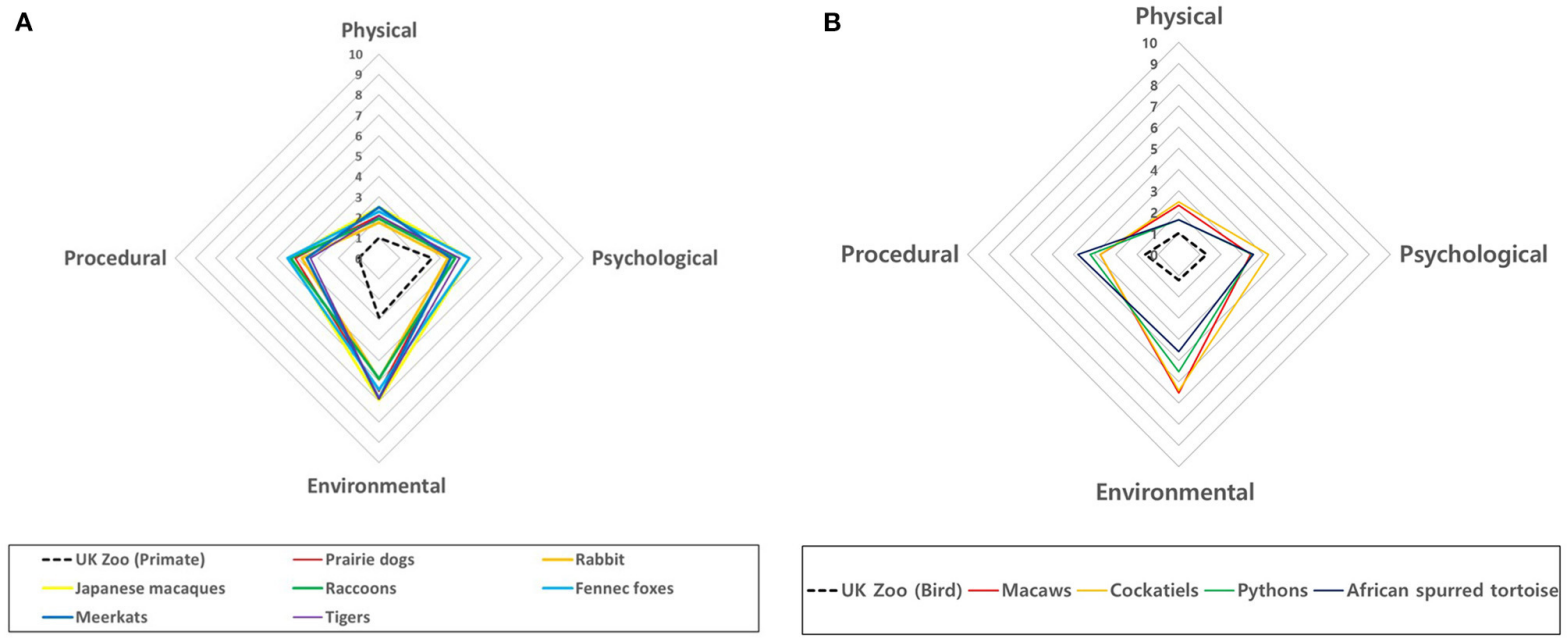
Modified Animal Welfare Assessment Grid for individuals of 11 species in South Korea (solid line) and all primates and all birds in the UK (dotted line). The shapes of the scores for the 11 Korean species were similar and larger than those for the UK primates and birds. **(A)** Comparison between UK primates and Korean mammals. **(B)** Comparison between UK birds and Korean birds and reptiles.

The AWAG scores of the four sections averaged for the 11 included species all showed similar shapes due to greater environmental (4.58–6.95), psychological (3.38–4.42), and procedural (3.54–4.78) scores compared to the physical scores (1.63–2.51) for all individuals. AWAG scores of Korean 11 zoo species showed higher in all sections than UK primates and birds, and the environmental score of Japanese macaques was vastly different compared with UK primates (Figure 1).

**Figure 2:**
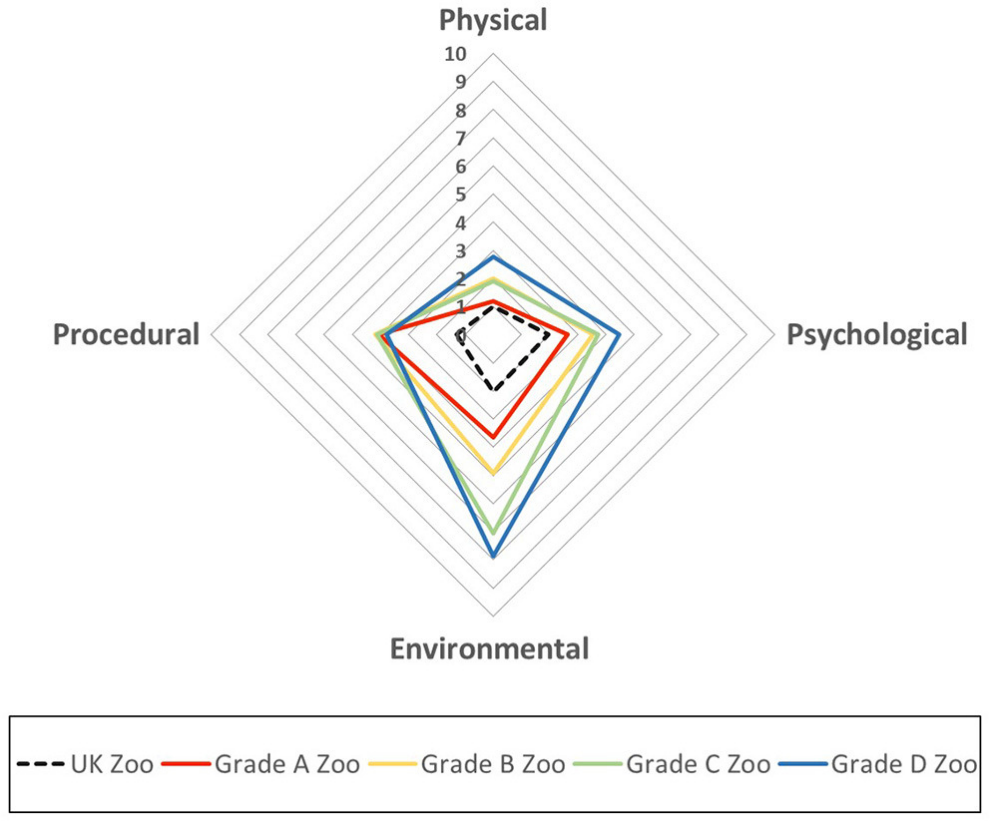
The average modified Animal Welfare Assessment Grid (AWAG) in four sections for zoo grades A, B, C, and D (solid line) compared to UK zoos (dotted line). The South Korean AWAG scores in each section for the four zoo grades were larger than those for the UK zoos.

The AWAG scores of each section increased from grade A to D. The procedural scores were similar, but the physical, psychological, and environmental scores were higher by 1.57, 1.82, and 4.21 points, respectively. The scores for the UK zoos were smaller than those for all grades of Korean zoos, even compared to grade A zoos (Figure 2).

The mean modified AWAG physical, psychological, environmental, and procedural scores were 2.11, 3.3, 4.89, and 2.77, respectively. The environmental score was approximately 1.5 to 3 points higher than those of the other sections (Table 3).

**Table 3:** Mean and 95% confidence intervals of four average AWAG scores among 16 zoos in South Korea.

**Physical section**	**Psychological section**	**Environmental section**	**Procedural section**
2.11 [1.77, 2.45]	3.31 [3.11, 3.51]	4.89 [4.51, 5.27]	2.77 [2.52, 3.02]

**Figure 3:**
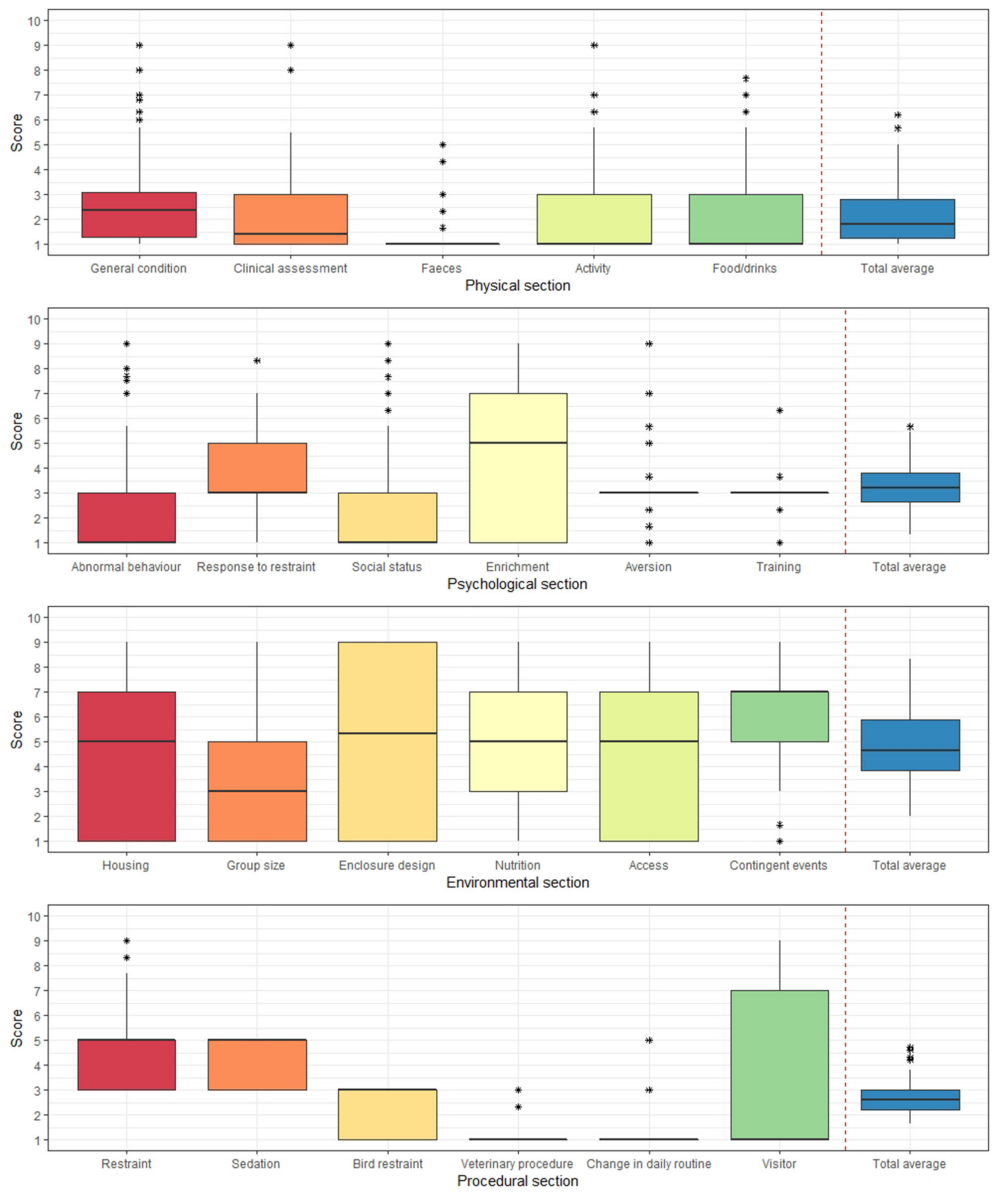
Box-and-whisker plots of the 23 modified Animal Welfare Assessment Grid (AWAG) scores of 11 species from 16 zoos in South Korea according to four sections (physical, psychological, environmental, and procedural). The scores were assessed from 1 to 10 points in the order of “very good” (1) to “worst” (10). Three groups of inspectors determined the scores for each animal and averaged the scores with very high inter-rater reliability. For each plot, the color represents the 23 AWAG indicators in four sections, the box represents the interquartile range (IQR), the whiskers represent 1.5 times the IQR range, the bold horizontal line represents the median, and each asterisk represents the outlier scores.

The mean physical score ranged from 1 to 3 (Figure 3). The fecal consistency score was very low, at 1 point for most zoos. The four scores in the psychological section (abnormal behavior, social status, aversion, and training) showed a low score distribution of 1–3. Responses to restraint showed a high score distribution from 3 to 5, while enrichment showed a large deviation between zoos, with a distribution from 1 to 7. We also observed high deviations of 4.5 points or more among all environmental scores, except for the contingent event score. The enclosure design score showed the largest deviation, from 1 to 9. The contingent event score had a high overall score of 5–7 points. In the procedural section, bird restraint, veterinary procedures, and changes in daily routine scores ranged from 1 to 3 points and restraint and sedation from 3 to 5 points. Visitor scores ranged from 1 to 7, with the largest difference in distribution among zoos.

The modified AWAG showed an IRR of 0.942 for all three inspectors' evaluations. The score for each inspector was high (>0.95) (Table 4).

**Table 4:** Inter-rater reliability (IRR) between each pair of inspectors and all three inspectors.

**Inspectors**	**Veterinarian & Zookeeper**	**Zookeeper & Researcher**	**Researcher & Veterinarian**	**All inspectors**
Inter-rater Reliability	0.963	0.965	0.971	0.942

*The Fleiss' Kappa was used to evaluate inter-rater reliability of AWAG scores among all three inspectors. The linearly weighted Cohen's Kappa was used to measure inter-rater reliabilities of each pair of inspectors*.

**Figure 4:**
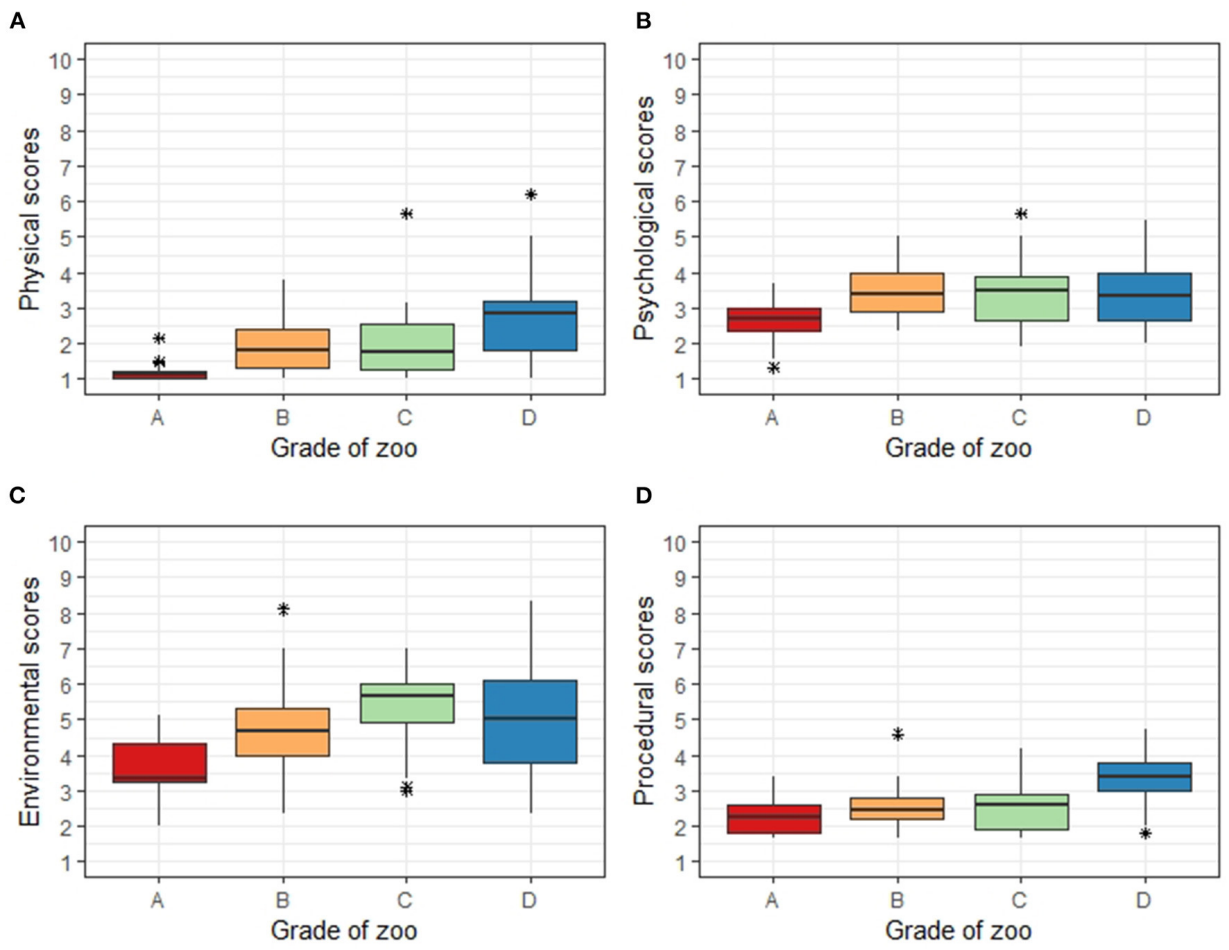
Box-and-whisker plots of averaged modified Animal Welfare Assessment Grid (AWAG) scores of 11 species from 16 zoos in South Korea according to zoo levels. The AWAG scores were assessed from 1 to 10 points in the order of “very good” (1) to “worst” (10). Three groups of inspectors determined the scores for each animal and averaged the scores with very high inter-rater reliability. The zoo levels were defined as **(A)** (AZA-accredited zoos), **(B)** (Korean Association of Zoos & Aquariums-certified zoos), **(C)** (large petting zoos, >50 species, >1,000 individuals, >3,000 m^2^), or **(D)** (small petting zoos). For each plot, the color represents the zoo levels, the box represents the interquartile range (IQR), the whiskers represent 1.5 times the IQR range, the bold horizontal line represents the median, and each asterisk represents the outlier scores.

Analysis of the differences in welfare evaluation scores by ANOVA revealed large differences in sections according to zoo grade (Table 5, Figure 4). Grade A zoos showed overall lower scores for all sections than the other zoos. Grade A zoos had lower physical scores than those of grades B and C zoos, whereas grade D zoos had higher physical scores than those for grades B and C zoos. Grade A zoos had lower psychological scores than those of grades B, C, and D zoos. Grade A zoos had lower environmental scores than those in grade B zoos, whereas grades C and D zoos had higher scores than those in grade B zoos. Grade A, B, and C zoos had similar procedural scores, with only grade D zoos showing relatively high values.

**Table 5:** Results of Kruskal–Wallis Analysis of variance (ANOVA) about the statistical difference of four AWAG section by the four level of zoos (A, B, C & D) in South Korea (α = 0.05).

**Physical section**	**Psychological section**	**Environmental section**	**Procedural section**
A < BC < D (*p*-value <0.001)	A < BCD (*p*-value = 0.00)	A < B < CD (*p*-value <0.001)	ABC < D (*p*-value <0.001)

We observed significant associations between the physical and psychological scores and between the environmental and procedural scores. In particular, the access score in the environmental section was positively correlated with the scores for general condition (1.04) and food/water intake (1.08) in the physical section. The restraint score in the procedural section was positively correlated with psychological scores, such as abnormal behavior (1.24), response to catching events (1.16), social status (1.22), and aversion to normal events (1.12). The veterinary procedure score in the procedural section was positively correlated with the general condition (1.14) and clinical assessment (1.20) scores in the physical section and the response to social status (1.18) score in the psychological section.

## Discussion

This study applied the modified AWAG to measure the welfare of 11 animal species in 16 Korean zoos. In a relatively short period (14 days), all 16 zoos were successfully assessed (within half a day or 1 day for each zoo). Despite the shortened and modified assessment format, the IRR of this tool was high, similar to that for the original AWAG (22). Therefore, the modified AWAG used in this study showed high usability and objectivity for index evaluations.

The modified AWAG used in this study identified differences in welfare levels according to the grade of Korean zoos. Grade A zoos, which were expected to have a high level of welfare according to AZA accreditation, showed an exceptionally low score distribution in all physical, psychological, environmental, and procedural sections, confirming appropriate zoo welfare. Zoo grades B, C, and D also differed by section, with differences in each score. Therefore, the modified AWAG was a good assessment tool to specifically confirm the welfare improvement of zoos (Figure 4).

In our study, compared to primates at UK zoos, mammal species in Korea had higher scores in all areas, except for the physical section. In particular, environment scores were vastly different for all animal species, except for tigers, with >6 points. This was also the case for birds and other animals. This reveals the differences between the UK, which has a licensing system and gives permission to inspect the welfare and environment of zoos, and Korea, which does not yet have such a system. These results in Korea were in contrast to the evaluation of UK zoos, in which >83% of the animals were graded as meeting the standards (Figure 1) (28). The scores for the procedural area for grade A zoos were similar to those in grades B, C, and D. This is because grade A zoos have a large number of visitors (more than 2 million visitors per year), animal training was newly introduced, and veterinary treatment was carried out. The animals in the grade C and D zoos had small numbers of visitors, the animals had been habituated to petting, and treatment was poor owing to the lack of veterinarians (28). All grades of Korean zoos had lower welfare compared to UK zoos; however, only primates and birds in two zoos in the UK were compared, which was not representative of the country overall. This may require an additional welfare investigation (Figure 2) (22).

We observed large differences in most environmental scores between zoos, with some zoos showing inadequate welfare. The enclosure design was the factor with the largest difference between zoos. Enclosure design maximizes the appearance of natural habitats, such as water puddles and mud baths, for animals to express their natural behaviors and provide hiding places. Inadequate enclosure design significantly impacts overall well-being, leading to deviations from natural behavior, self-mutilation, or coprophagia (29). Therefore, Korean zoos must prioritize their enclosure designs (Figure 3).

The enrichment score in the psychological section also showed large differences between zoos. For example, the meerkat enclosure of a grade A zoo provided more than five types of enrichment items, including spreading insects, food puzzles, and enrichment toys. However, in one grade D zoo, the meerkats were in a glass-walled space on a cement floor without any environmental enrichment. In addition, newly hired managers were unaware of the need for enrichment; therefore, the score of welfare evaluations were lowest in these zoos. This difference eventually resulted in serious stereotypical behaviors in the meerkats at those zoos. In Korea, while behavioral enrichment was introduced in zoos in 2003, it has not been implemented in the most of zoos (15). In the procedural section, visitor scores also differed significantly between zoos. The number of visitors affects the welfare of sensitive animals such as birds and mammals. The greater the number of visitors, the greater the noise generated, which affects mammalian behavior and physiology. In addition, Korean zoos have continuous monitoring, such as measuring the stress of animals in zoos with poor visitor ratings, as 20 zoos within the KAZA attract more than 20 million visitors per year. The stress of animals must be considered, such as improving visitor behavior, through increased campaigns or education (30).

Evaluation of animal welfare in Korean zoos using the modified AWAG revealed how the differences between the environmental and procedural scores were related to the physical and psychological scores of the animals through correlation analyses between variables. In this study, the access score was positively correlated with the general condition and food/water intake and was an indicator of the degree to which animals could freely use their shelters and the extent to which they were confined to a non-stimulating space. Environmental accessibility is important to increase the selection and utilization of available space for animals, as noted in previous studies on its effect on the welfare of chimpanzees (31). In the present study, the access score was positively correlated with the general condition (1.04) and food and drink (1.08). Decreased accessibility leads to a poorer general condition, which will reduce its food and water intake (Table 6).

**Table 6:** Estimates and 95% confidence intervals of odds ratio of physical AWAG factors, or any of environmental and procedural factors (α = 0.05).

**Physical section**		**General condition**	**Clinical assessment**	**Activity**	**Food and drinks**
Environmental section	Enclosure design				0.95 [0.92, 0.99]
	Access	1.04 [1.00, 1.08]			1.08 [1.03, 1.12]
	Contingent events			1.08 [1.03, 1.14]	1.11 [1.05, 1.17]
Procedural section	Restraint			1.19 [1.10, 1.28]	
	Veterinary procedure	1.14 [1.00, 1.28]	1.20 [1.04, 1.37]		

*Only results derived were indicated*.

We also analyzed the relationships between psychological AWAG factors and environmental or procedural factors (Table 7). The most striking factor was the restraint score in the procedural section, which showed many positive correlations with the other welfare scores. In general, the higher the restraint score (i.e., no restraint training), the more abnormal the behavior, the greater the response to a catching event, the worse the social status, and the stronger the aversion to normal events. While training in zoos was previously used only in circuses or zoo performances, it has recently been recognized as a good way to improve animal welfare and health and its use is spreading in many zoos (32). Although whether medical training can improve animal welfare is controversial, the results of this study represent a major improvement in animal welfare as it shows the potential positive effect on the physical and psychological effects of animals (33).

**Table 7:** Estimates and 95% confidence intervals of odds ratio of psychological AWAG factors or any of environmental and procedural factors (α = 0.05).

**Psychological section**		**Abnormal behavior**	**Response to catching event**	**Social status**	**Enrichment**	**Aversion to ‘normal' events**
Environmental section	Group size			1.10 [1.06, 1.15]		
	Nutrient				1.04 [1.01, 1.07]	
Procedural section	Restraint	1.24 [1.00, 1.57]	1.16 [1.10, 1.23]	1.22 [1.11, 1.34]		1.12 [1.04, 1.19]
	Sedation			0.81 [0.71, 0.93]		
	Veterinary procedure			1.18 [1.04, 1.33]		

*Only results derived were indicated*.

Although many attempts have been made to evaluate animal welfare in zoos, this evaluation remains complicated and difficult (34). This study measured multiple zoos only once. Therefore, this protocol will be useful for assessing institutions and welfare factors within a short period through the evaluation of zoos at the national level. However, there are limitations in identifying changes in animal welfare over time and evaluating the impact of improvements in welfare factors. Subsequent research should measure long-term changes in welfare through continuous measurement, as in existing AWAG research (22). However, since the measurement of all scores may have practical limitations, this aspect can be supplemented by identifying an indicator that varies over time and by periodically inspecting only this relevant indicator or by continuously monitoring welfare evaluation by season. In terms of individual zoos, it can be helpful to improve relatively poor welfare factors that are relatively poor and to improve the welfare of individual zoos through continuous monitoring of low-scoring items through managerial education. A few scores that required continuous monitoring and improvement cycles, including fecal consistency, aversion to normal events, training, veterinary procedures, and changes in routine, did not show relatively disparate differences and thus may require additional research.

The modified AWAG is an efficient assessment tool that can be conveniently applied in the field. It can be used to perform evaluations in a short time and showed a high IRR and high objectivity (Table 4). The modified AWAG allowed the identification of differences in welfare at different zoo grade levels in Korea. Among these, housing, enclosure design, and enrichment were environmental factors that required improvement. Furthermore, the identification of statistical correlations between the scores revealed which environmental or procedural sections could help improve physical and psychological scores.

Our results showed that this is an efficient, reliable, and objective zoo evaluation method that may be a good option when implementing a licensing system in a country like Korea, which lacks inspectors with sufficient knowledge and experience. In addition, this method can be applied at the national level if a more objective welfare evaluation is required in countries with a licensing system. Continuous use of the modified AWAG is an objective method that sets the direction for improvement in zoo welfare. Improvements should be periodically monitored, for which we expect continued use of this tool.

## Data Availability Statement

The original contributions presented in the study are included in the article/Supplementary Material, further inquiries can be directed to the corresponding author.

## Ethics Statement

Ethical review and approval was not required for the animal study because only observation study, not required in Korea.

## Author Contributions

SM: research design planning, field research (welfare inspector) and manuscript writing. HK: research design planning and field research (welfare inspector). KL: research design planning, data analysis and manuscript writing. SK: research design planning and manuscript writing. JH: principle investigator. All authors contributed to the article and approved the submitted version.

## Funding

This study was funded under contract/grant number 2020-330 from the Study of the Operation and Management of the Zoo Permit Standards Project, Ministry of Environment of the Republic of Korea.

## Conflict of Interest

The authors declare that the research was conducted in the absence of any commercial or financial relationships that could be construed as a potential conflict of interest.

## Publisher's Note

All claims expressed in this article are solely those of the authors and do not necessarily represent those of their affiliated organizations, or those of the publisher, the editors and the reviewers. Any product that may be evaluated in this article, or claim that may be made by its manufacturer, is not guaranteed or endorsed by the publisher.
